# A novel BMPR2 mutation in a patient with heritable pulmonary arterial hypertension and suspected hereditary hemorrhagic telangiectasia: A case report

**DOI:** 10.1097/MD.0000000000021342

**Published:** 2020-07-31

**Authors:** Fanhao Ye, Wenbing Jiang, Wei Lin, Yi Wang, Hao Chen, He Zou, Shiwei Huang, Ning Zhu, Sisi Han

**Affiliations:** Department of Cardiology, Wenzhou People's Hospital, Wenzhou Third Clinical Institute Affiliated with Wenzhou Medical University, Wenzhou, Zhejiang, China.

**Keywords:** bone morphogenetic protein receptor 2mutation, hereditary hemorrhagic telangiectasia, heritable pulmonary arterial hypertension, nonsense mutation

## Abstract

**Rationale:**

*BMPR2* mutation is the most common cause of heritable pulmonary arterial hypertension (HPAH), but rare in hereditary hemorrhagic telangiectasia (HHT). *ACVRL1*, *ENG* and *SMAD4* are the most common gene mutations reported in HPAH with HHT.

**Patient concerns:**

We report a 11-year-old boy with a definite diagnosis of pulmonary hypertension and suspected HHT with recurrent epistaxis. The results of gene detection showed that there was a nosense mutation in *BMPR2*. The results of gene detection of *ACVRL1, ENG* and *SMAD4* were normal.

**Diagnoses:**

Heritable pulmonary arterial hypertension with suspected hereditary hemorrhagic telangiectasia.

**Interventions:**

Patient was treated with ambrisentan 2.5 mg qd. About a month later, the patient developed massive gastrointestinal bleeding and sudden convulsions. The patient's vital signs were stable after symptomatic treatment.

**Outcomes:**

After discharging from hospital, the patients continued to take ambrisentan. No epistaxis or gastrointestinal bleeding was found in one month of follow-up, but the symptoms of chest tightness were not significantly alleviated.

**Lessons:**

*BMPR2* with a nonsense mutation is more likely to cause HPAH with HHT and are more likely to be life-threatening.

## 1 Introduction

Heritable pulmonary arterial hypertension (HPAH) is a group of diseases characterized by persistent increase of pulmonary vascular resistance and progressive right heart failure caused by genetic factors, which includes clinically sporadic idiopathic PAH with germline mutations and clinical familial cases with or without identified germline mutations.^[1]^ The bone morphogenetic protein receptor 2 (*BMPR2*) gene mutation is an important pathogenic factor of HPAH.^[2,3]^ It exists in around 70% to 80% of families with PAH and 10% to 20% of IPAH cases.^[4]^

Hereditary hemorrhagic telangiectasia (HHT) is usually associated with mutations in endoglin (*ENG*), activin receptor-like kinase 1 *(ACVRL1*), mothers against decapentaplegic homolog 4 (*SMAD4*), etc. Only 1 case reported that *BMPR2* gene analysis is indicated in patients affected with both HHT and HPAH.^[5]^ Here we report an 11-year-old boy diagnosed with HPAH and suspected HHT, which may be associated with a novel nonsense mutation of *BMPR2*.

## 2 Case report

**Figure 1:**
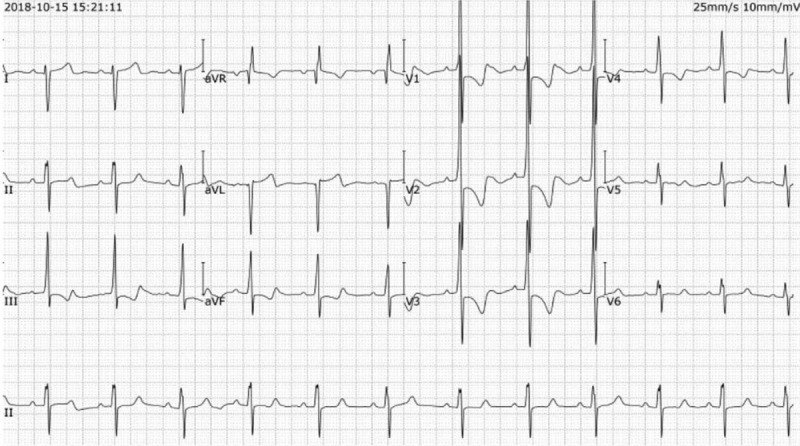
Electrocardiograph revealed a sinus rhythm, and right axis deviation reached + 132°.

**Figure 2:**
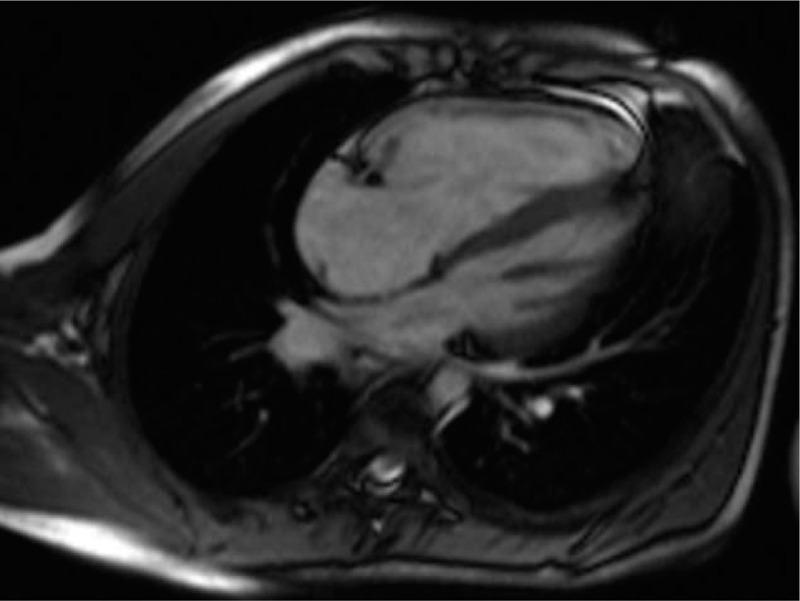
Cardiac magnetic resonance revealed a enlargement of right atrium and right ventricle.

A 11-year-old boy was admitted to our outpatient department on October 15, 2018, with chest tightness after exercise for 5 years, aggravated in half a month, and repeated hemorrhinia for 2 years. The patient had a history of syncope 1 year ago. His grandmother had a history of pulmonary hypertension. On admission examination, his heart rate was 78 beats/min, his blood pressure was 102/65 mm Hg, and his SpO_2_ was 96%. The heart sounds were regular, and no murmur was detected. No obvious abnormalities were observed in the rest of the physical examinations. Blood tests noted normal hemoglobin (146 g/L, normal 130–175), increased N-terminal pro-brain natriuretic peptide (NT-ProBNP, 643pg/ml, normal <125), increased total bilirubin (30 μmol/L, normal 3.4–21.1), increased direct bilirubin (10.1 μmol/L, normal < 6.8), increased indirect bilirubin (20.6 μmol/L, normal < 3.1–17.0), and increased alkaline phosphatase (324U/L, normal 45–125). Electrocardiograph (ECG) revealed a sinus rhythm, and right axis deviation reached + 132° (Fig. 1). Transthoracic echocardiography revealed a right atrial dimension of 40 mm, a right ventricular dimension of 30 mm, a left atrial dimension of 28 mm, a left ventricular end-diastolic dimension of 43 mm, a left ventricular end-systolic dimension of 30 mm, a left ventricular ejection fraction of 73%, a tricuspid annular plane systolic excursion of 15 mm, a maximum tricuspid regurgitation velocity, and a pulmonary artery systolic pressure of 91 mm. Cardiac magnetic resonance revealed a enlargement of right atrium and right ventricle (Fig. 2). Cardiopulmonary exercise test showed that the overall functional status of cardiopulmonary motor function, the effectiveness of oxygen uptake ventilation and nitrogen dioxide ventilation were significantly limited. Computed tomographic pulmonary angiography (CTPA) showed enlargement of right ventricle, dilatation of main pulmonary artery, changes of pulmonary hypertension, and diffuse small patches of light ground glass opacity in both lungs.

The patient underwent right cardiac catheterization, acute pulmonary vasodilator testing (AVT) and pulmonary angiography on October 29, 2018. Details are as follows: (1) Baseline blood pressure was 115/54/74 mm Hg, pulmonary arterial pressure (PAP) was 95/40/59 mm Hg, pulmonary artery wedge pressure was 8/7/6 mm Hg, cardiac output was 4.23L/min, cardiac index was 2.9L/min/m^2^, and pulmonary vascular resistance was 12.53 Wood U, (2) PAP was 109/32/60 mm Hg after inhalation of vantamivir (AVT negative), (3) Pulmonary angiography showed dilatation of left and right pulmonary arteries, no obvious stenosis, filling defect or occlusive lesions, no decrease or absence of distal segmental perfusion, and the perfusion velocity was acceptable.

Later, we used high-throughput gene sequencing technology to detect all exon genes for the patient. Genetic tests revealed that the patient carried a non-sense mutation of BMPR2, c. 1452G > A, which resulted in the termination of BMPR2 protein at 484 position and significantly affected the function of the protein (Table 1). Mutation analysis of *ACVRL1*, *ENG,* and *SMAD4* was normal.

**Table 1:**
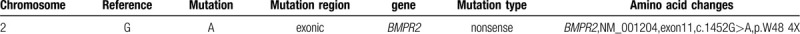
Rare mutation of *BMPR2* in this patient.

According to the symptoms, signs and examination results, we diagnosed familial pulmonary arterial hypertension and suspected hereditary hemorrhagic telangiectasia. The patient was discharged from hospital on October 28, 2018, and was treated with ambrisentan 2.5 mg qd.

On November 26, 2018, the patient had a large amount of black stool. The stool was hard and normal in shape, and there was little blood on the surface. The patient had abdominal discomfort and no bleeding and ecchymosis on the skin. At 4 am on November 28, 2018, the patient woke up in sleep, followed by convulsion, characterized by sudden loss of consciousness, eyeball upturn, sudden generalized and ankylosing facial and extremity muscle convulsions, cyanosis of lips, incontinence of urine and feces, and self-remission after 30 seconds. The patient was immediately admitted to the emergency department. Blood tests noted decreased hemoglobin (62 g/L, normal 130–175), increased NT-ProBNP (1010pg/ml, normal < 125), increased creatine kinase (721U/L, normal 55–170), increased lactate dehydrogenase (247U/L, normal 135–225). Faecal occult blood test was positive. There were no significant changes in echocardiographic data. Short-range video electroencephalogram was normal. No obvious abnormalities were found in cranial magnetic resonance imaging and magnetic resonance angiography. We planned to have further gastrointestinal endoscopy, but the patient's family refused. Acute gastrointestinal bleeding and convulsions caused by acute ischemic-hypoxic encephalopathy were considered in the diagnosis. Therapeutically, omeprazole was given to inhibit gastric acid secretion, tranexamic acid and etamsylate were used to stop bleeding, and suspended red blood cells were infused. On December 2, 2018, hemoglobin was 104 g/L, and fecal occult blood test was negative. During the follow-up for the next month, the patient had no black stool, no hemorrhinia, and no significant changes in chest tightness.

## 3 Discussion

In view of the hereditary pulmonary arterial hypertension in this patient, the mutation of BMPR2 gene was considered. The 1452 base G of exon 11 of *BMPR2* gene in this patient was replaced by base A, which resulted in the change of tryptophan at position 484 to termination code, that is nonsense mutation.

*BMPR2* gene belongs to the transforming growth factor-β (TGF-β) receptor family. TGF-β superfamily signal transduction pathway can inhibit the proliferation and induce apoptosis of vascular smooth muscle and endothelial cells. BMP binds to BMPR2 and is phosphorylated, then activates BMPR1 to form a complex. The serine/threonine disability in Gly-Ser region of BMPR1 is phosphorylated by BMPR2, followed by the phosphorylation of Smad1, 5, 8 proteins, which dissociates with BMPR1. Two Smad1 and Smad4 bind to the nucleus and bind to specific transcriptional promoters or repressors, which act on transcriptional regulators to inhibit cell proliferation and promote cell apoptosis.^[6]^ Therefore, it is presumed that abnormal BMPR2, as a cell surface receptor, may disrupt the signal pathway mediated by BMPR2, leading to uncontrolled proliferation of pulmonary artery wall cells, thus causing pulmonary arterial hypertension.^[7]^

HHT is an autosomal dominant vascular disorder. The disease is caused by pathogenic mutations of either *ACVRL1* or *ENG*,^[8–11]^ and, rarely, Smad 4, which is also associated with juvenile polyposis.^[3]^ The patient had recurrent epistaxis and massive gastrointestinal hemorrhage. CTPA showed diffuse small patches of light ground glass opacity in both lungs. According to the diagnostic criteria of HHT,^[12]^ the presence of suspicious hereditary hemorrhagic telangiectasia was considered.

BMP9 signaling in endothelial cells is a key mechanism in the pathogenesis of HHT.^[5]^ It is a special ligand for ALK1, ENG, BMPRII, and the type-IIA activin receptor (ActRIIA) which can maintain BMP9 signaling, so that *BMPR2* mutations do not usually lead to overt HHT.^[13]^ However, a *BMPR2* mutation[c.1297C>T (p.Q433X)] in which ActRIIA cannot complement BMPRII function has been reported, and leads to the manifestation of HHT in HPAH patients.^[5]^ Our patient also had nonsense mutations in *BMPR2*, so we suspect that premature termination of translation of the *BMPR2* gene may result in greater BMPRII dysfunction. This may seriously affect the BMP9 signal, even if ActRIIA works.

## 4 Conclusion

The novel BMPR2 mutation we report is extremely dangerous because it not only causes pulmonary hypertension, but also manifests as HHT. In this patient, hemorrhagic anemia aggravates the decrease of cardiac output caused by pulmonary hypertension, leading to severe hypoxemia and acute ischemic-hypoxic encephalopathy. Through active treatment, although the vital signs of the patient has been stabilized, the therapeutic effect of ambrisentan on PHA is not ideal at present. The patient needs a longer follow-up to assess the efficacy of antibiotics and the long-term prognosis of this *BMPR2* mutation.

## Acknowledgments

The authors thank the family for participating and supporting this study.

## Author contributions

**Data curation:** Fanhao Ye, Wenbing Jiang, Ning Zhu, Sisi Han.

**Investigation:** Fanhao Ye, Wei Lin, Hao Chen, He Zou, Shiwei Huang.

**Supervision:** Fanhao Ye, Yi Wang.

**Writing – original draft:** Fanhao Ye, Wenbing Jiang.

**Writing – review & editing:** Fanhao Ye, Yi Wang.

## References

[1] Galiè N Hoeper MM Humbert M Guidelines for the diagnosis and treatment of pulmonary hypertension: the Task Force for the Diagnosis and Treatment of Pulmonary Hypertension of the European Society of Cardiology (ESC) and the European Respiratory Society (ERS), endorsed by the International Society of Heart and Lung Transplantation (ISHLT). Euro Heart J 2009;30:2493–537. 10.1093/eurheartj/ehp297 19713419

[2] Machado RD Aldred MA James V Mutations of the TGF-( type II receptor BMPR2 in pulmonary arterial hypertension. Human mutation 2006;27:121–32. 16429395 10.1002/humu.20285

[3] Machado RD Eickelberg O Elliott CG Genetics and genomics of pulmonary arterial hypertension. J AmColl Cardiol 2009;54:S32–42. 10.1016/j.jacc.2009.04.015 PMC3725550 19555857

[4] Evans JD Girerd B Montani D BMPR2 mutations and survival in pulmonary arterial hypertension: an individual participant data meta-analysis. Lancet Respir Med 2016;4:129–37. 26795434 10.1016/S2213-2600(15)00544-5 PMC4737700

[5] Rigelsky CM Jennings C Lehtonen R BMPR2 mutation in a patient with pulmonary arterial hypertension and suspected hereditary hemorrhagic telangiectasia. Am J of Med Genet A 2008;146:2551–6. 10.1002/ajmg.a.32468 18792970

[6] Derynck R Zhang YE Smad-dependent and Smad-independent pathways in TGF-beta family signalling. Nature 2003;425:577–84. 14534577 10.1038/nature02006

[7] McLaughlin VV McGoon MD Pulmonary arterial hypertension. J Circulation 2006;114:1417–31. 10.1161/CIRCULATIONAHA.104.503540 17000921

[8] Chaouat A Coulet F Favre C Endoglin germline mutation in a patient with hereditary haemorrhagic telangiectasia and dexfenfluramine associated pulmonary arterial hypertension. Thorax 2004;59:446–8. 15115879 10.1136/thx.2003.11890 PMC1746994

[9] Harrison RE Flanagan JA Sankelo M Molecular and functional analysis identifies ALK-1 as the predominant cause of pulmonary hypertension related to hereditary haemorrhagic telangiectasia. J Med Genet 2003;40:865–71. 14684682 10.1136/jmg.40.12.865 PMC1735342

[10] Harrison RE Berger R Haworth SG Transforming growth factor-receptor mutations and pulmonary arterial hypertension in childhood. Circulation 2005;111:435–41. 15687131 10.1161/01.CIR.0000153798.78540.87

[11] Mache CJ Gamillscheg A Popper HH Early-life pulmonary arterial hypertension with subsequent development of diffuse pulmonary arteriovenous malformations in hereditary haemorrhagic telangiectasia type 1. Thorax 2008;63:85–6. 18156574 10.1136/thx.2007.076109

[12] Shovlin CL Guttmacher AE Buscarini E Diagnostic criteria for hereditary hemorrhagic telangiectasia (Rendu Osler Weber syndrome). Am J Med Genet 2000;91:66–7. 10751092 10.1002/(sici)1096-8628(20000306)91:1<66::aid-ajmg12>3.0.co;2-p

[13] David L Mallet C Mazerbourg S Identification of BMP9 and BMP10 as functional activators of the orphan activin receptor-like kinase 1 (ALK1) in endothelial cells. Blood 2007;109:1953–61. 17068149 10.1182/blood-2006-07-034124

